# Extracellular Vesicles as a Potential Biomarker of Pulmonary Arterial Hypertension in Systemic Sclerosis

**DOI:** 10.3390/ph18020259

**Published:** 2025-02-14

**Authors:** Stelvio Tonello, Davide D’Onghia, Annalisa Di Ruscio, Silvia Maria Mora, Federica Vincenzi, Giulia Caria, Alessia Fracchia, Nicole Vercellino, Benedetta Bussolati, Adele Tanzi, Manuela Rizzi, Rosalba Minisini, Daniele Sola, Massimo Scacchi, Stefania Mai, Mario Pirisi, Carlo Smirne, Elena Grossini, Vincenzo Cantaluppi, Cristoforo Comi, Giuseppe Murdaca, Donato Colangelo, Pier Paolo Sainaghi

**Affiliations:** 1 Department on Translational Medicine, Università del Piemonte Orientale, 28100 Novara, Italy; stelvio.tonello@med.uniupo.it (S.T.); davide.donghia@uniupo.it (D.D.); 20019360@studenti.uniupo.it (S.M.M.); 20028271@studenti.uniupo.it (F.V.); 20033138@studenti.uniupo.it (G.C.); 20033432@studenti.uniupo.it (A.F.); nicole.vercellino@uniupo.it (N.V.); rosalba.minisini@med.uniupo.it (R.M.); mario.pirisi@uniupo.it (M.P.); carlo.smirne@med.uniupo.it (C.S.); pierpaolo.sainaghi@uniupo.it (P.P.S.); 2 Cancer Research Institute, Beth Israel Deaconess Medical Center, Boston, MA 02215, USA; adirusci@bidmc.harvard.edu; 3 Harvard Medical School Initiative for RNA Medicine, Harvard Medical School, Boston, MA 02115, USA; 4 Molecular Biotechnology Center “Guido Tarone”, Department of Molecular Biotechnology and Health Sciences, Università di Torino, 10125 Torino, Italy; benedetta.bussolati@unito.it (B.B.); adele.tanzi@unito.it (A.T.); 5 Human Anatomy Laboratory, Department of Health Sciences, Università del Piemonte Orientale (UPO), 28100 Novara, Italy; 6 Laboratory of Metabolic Research, IRCCS Istituto Auxologico Italiano, 28824 Oggebbio, Italy; massimo.scacchi@unimi.it (M.S.); s.mai@unimi.it (S.M.); 7 Department of Clinical Sciences and Community Health, Università di Milano, 20122 Milano, Italy; 8 Department of Internal Medicine and Rheumatology Unit, AOU Maggiore della Carità, 28100 Novara, Italy; 9 Laboratory of Physiology, Department of Translational Medicine, Università del Piemonte Orientale (UPO), 28100 Novara, Italy; elena.grossini@uniupo.it; 10 Nephrology Unit, Department of Translational Medicine, Università del Piemonte Orientale (UPO), 28100 Novara, Italy; vincenzo.cantaluppi@uniupo.it; 11 Neurology Unit, Department of Translational Medicine, Università del Piemonte Orientale (UPO), 28100 Novara, Italy; cristoforo.comi@uniupo.it; 12 Department of Internal Medicine, University of Genova, 16132 Genova, Italy; giuseppe.murdaca@unige.it; 13 Allergology and Clinical Immunology Unit, San Bartolomeo Hospital, 19038 Sarzana, Italy; 14 Pharmacology, Department of Health Sciences, Università del Piemonte Orientale (UPO), 28100 Novara, Italy; donato.colangelo@med.uniupo.it; 15 CAAD-Center for Autoimmune and Allergic Diseases and IRCAD-Interdisciplinary Research Center for Autoimmune Diseases, Università del Piemonte Orientale (UPO), 28100 Novara, Italy

**Keywords:** extracellular vesicles, systemic sclerosis, pulmonary hypertension, pulmonary fibrosis, autoimmunity

## Abstract

**Introduction:** Pulmonary arterial hypertension (PAH) and interstitial lung disease (ILD) are severe complications of patients with systemic sclerosis (SSc). Currently, there are a few tests for early identification of these conditions, although they are invasive and time-consuming. Extracellular vesicles (EVs) offer a promising possibility for gathering information on tissue health. This study aims to characterize EVs in cases of systemic sclerosis complicated by pulmonary hypertension and pulmonary fibrosis. **Methods:** A cohort of 58 patients with SSc was evaluated, including 14 with pulmonary hypertension, 17 with pulmonary fibrosis, and 27 without complications. Additionally, 11 healthy subjects, matched for sex and age, served as a control group. EVs were characterized by using a MACSplex kit to analyze the expression of 37 membrane markers. **Results:** After the overall analysis, we show that EVs from SSc patients had higher expression of CD146, CD42a, and CD29 (*p* = 0.03, *p* = 0.02 and *p* = 0.05) but lower expression of HLA-ABC with respect to the control patients (*p* = 0.02). Multivariate analyses demonstrated that only CD42a has a significant association with the disease (*p* = 0.0478). In group comparative analyses (PAH, ILD, uncomplicated systemic sclerosis (named SSc no PAH no ILD), and controls), CD3 and CD56 were higher in PAH patients, with respect to the controls, ILD, and the group SSc no PAH no ILD (CD3: *p* = 0.01, *p* = 0.003, *p* = 0.0005; CD56: *p* = 0.002, *p* < 0.0001, *p* = 0.0002). HLA-DR showed higher expression in PAH patients with respect to ILD patients (*p* = 0.02), CD25 showed higher expression in PAH patients with respect uncomplicated SSc (*p* = 0.02), and CD42a showed higher expression in PAH patients with respect to the controls (*p* = 0.03); nevertheless, multivariate analyses demonstrated that only CD3 retained its association with PAH. **Conclusions:** The expression of CD42a, a platelet-derived marker indicating endothelial damage, suggests its potential to provide information on the state of the microcirculation in systemic sclerosis. The higher expression of CD3 on the surface of the EVs in PAH patients might indicate increased T-cell activity in tissues, with a possible association with the development of pulmonary hypertension.

## 1. Introduction

Systemic sclerosis (SSc) is a rare chronic autoimmune connective tissue disease characterized by profound alterations in both the innate and adaptive immune responses. The hallmark features of SSc include widespread microvascular damage and fibrosis affecting the skin and internal organs [1]. The immune system, with a notable involvement of B and T cells, assumes a pivotal role in the inflammatory pathogenesis of SSc [2]. Upon the onset of SSc, activation of naïve T cells occurs, necessitating initial activation by an antigen-presenting cell, followed by co-stimulation through a non-antigen-specific co-stimulatory signal [3]. Defective regulation in this co-stimulatory signal and the imbalance of proinflammatory factors presents a potential pathway for the development of SSc [4]. The pathogenetic course of SSc leads to endothelial damage at multiple levels, characterized by disruptions in vascular tone regulation and the presence of proliferative vasculopathy [5]. SSc exhibits an elevated inflammatory profile, often linked to the proliferative response of fibroblasts, which ultimately lead to fibrosis [6,7].

One of the main sites of fibrotic involvement is the lung. The interstitial lung disease of SSc is framed in the context of connective tissue diseases (CTDs) associated with interstitial lung disease (ILD). This is a heterogeneous group of clinical entities characterized by the dysfunction of resident cells and widespread infiltration of inflammatory cells, leading to excessive matrix production in the alveoli or interstitial space [8]. Patients with interstitial lung disease, regardless of its origin, typically manifest with three distinct features: a restrictive ventilatory defect, reduced DLCO (diffusing capacity of the lung for carbon monoxide), and observable alveolar and interstitial changes on high-resolution computed tomography (HRCT) [9]. The pathogenesis remains intricate and not fully elucidated. A prominent hypothesis posits a pathological process initiated by acute repetitive alveolar lesions, leading to chronic interstitial inflammation, with activated fibroblasts playing a central role [10].

Another life-threatening complication of systemic sclerosis is pulmonary hypertension. This pathology falls into the type 1 pulmonary hypertension group, i.e., pulmonary arterial hypertension, and recognizes peculiar pathogenetic processes that overlap with those of connective tissue disease [11]. Pulmonary arterial hypertension (PAH) stands as a progressive condition impacting the pre-capillary pulmonary vascular bed, culminating in an escalation of pulmonary vascular resistance and consequential right ventricular failure, marked by a notable mortality rate [12]. This condition represents a formidable complication observed in diverse connective tissue diseases (CTDs), notably systemic sclerosis (SSc), mixed connective tissue disease (MCTD), and instances of SSc overlapping with other CTDs [13]. The timely identification of SSc-PAH remains a difficult challenge, given that PAH typically manifests with minimal symptoms in its initial stages. Since the prompt initiation of an efficacious therapeutic regimen ameliorates the prognosis of individuals afflicted by PAH, SSc patients undergo routine surveillance and screening for this complication [14]. The DETECT algorithm, a two-step screening protocol, stands as the prevalent modality for this purpose, combining clinical, laboratory, and instrumental parameters [15]. Notwithstanding the high sensitivity exhibited by the evidence-based screening DETECT algorithm (96%), an indispensable criterion for any screening tool, its specificity remains suboptimal (48%), necessitating a considerable number of unwarranted invasive determinations of pulmonary pressure via right heart catheterization (RHC) [16]. Considering this inherent limitation, there is an urgent requirement for innovative biomarkers capable of refining the risk stratification of PAH within the cohort of CTD patients [17].

Extracellular vesicles (EVs) are categorized into three primary subtypes based on size and biogenesis. Apoptotic bodies, the largest vesicles (500–1500 nm), are released by apoptotic cells. Macrovesicles/microparticles, large vesicles (120–500 nm), originate from the plasma membrane through budding, and exosomes, small vesicles (50–150 nm), originate from the endosomal compartment [18]. However, due to overlapping sizes among subtypes, current isolation procedures often yield either small-sized vesicles (ssEVs) or large-sized vesicles (lsEVs), posing a challenge in distinguishing the vesicles based on their origin [18]. EVs function as carriers for a diverse array of bioactive factors, including proteins, lipids, DNA, mRNA, and miRNAs, thereby playing pivotal roles in intercellular communication. They have been isolated from various biological fluids, spanning from ascitic fluid to urine, and those circulating in the bloodstream. Despite significant progress in understanding the role of EVs in oncology, their role in autoimmune diseases, including SSc, remains incompletely understood. While EVs hold promise as potential biomarkers for pulmonary hypertension associated with rheumatic diseases, their specific contribution remains to be fully elucidated [19].

The aim of the present study is to characterize the molecular profile of plasma extracellular vesicles in different subjects—SSc patients without lung involvement (SSc no PAH no ILD), patients with the lung pathologies ILD (interstitial lung disease) and pulmonary arterial hypertension (PAH), and healthy controls (controls)—and to identify differences in vesicular profiles among these groups that could potentially establish extracellular vesicles as biomarkers for clinical classification at diagnosis and during follow-up assessments.

## 2. Results

For the present study, 58 patients (100% females), with a median age of 65 [20,21] years and an SSc diagnosis, were recruited, the general features of the study population are summarized in Supplementary Table S1. Furthermore, 11 healthy subjects were selected as the control group of our study, matched by age and sex. The cohort study population was divided into three subgroups based on the features of the disease: 14 (24%) patients were affected by pulmonary arterial hypertension (PAH) as the main complication of the disease, and 17 (29%) patients by ILD, while 27 (47%) patients were without PAH or ILD (SSc no PAH no ILD). The demographic, clinical, and laboratory features of the population divided by subgroups are presented in Table 1 and Table 2.

### 2.1. Extracellular Vesicle Surface Epitopes Associated with SSc

**Figure 1:**
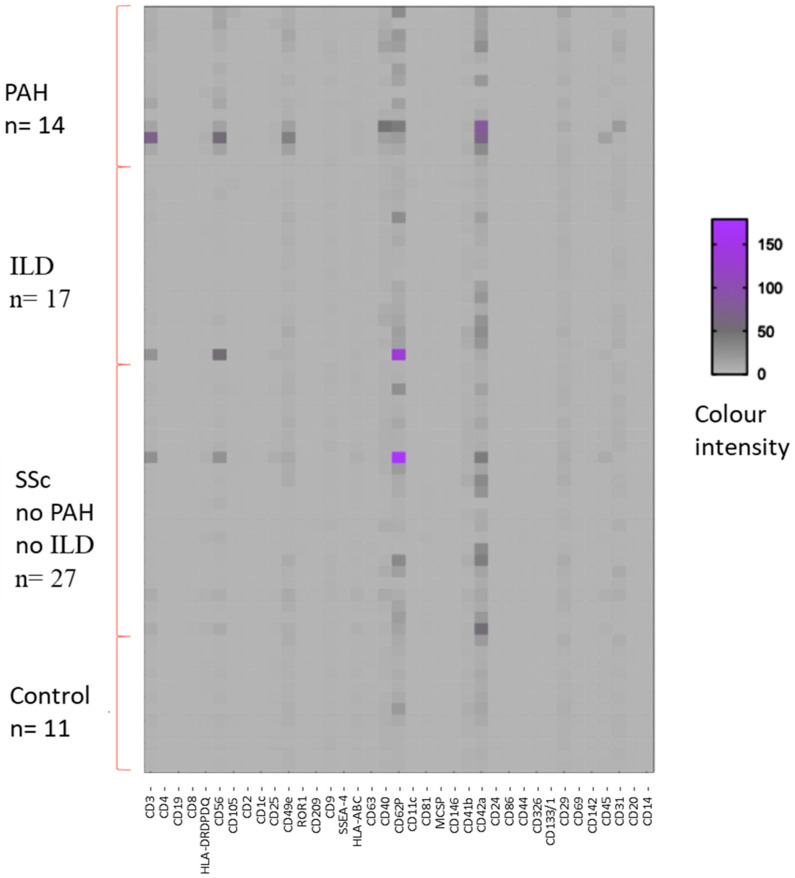
Heat map of flow cytometry results that illustrates the correlation between the analyzed markers and the study population divided by disease subgroups and controls. Patients are represented on the ordinate axis, while the entire panel of markers is represented on the abscissa axis. PAH = pulmonary arteriolar hypertension; ILD = interstitial lung disease; SSc no PAH no ILD = patients with SSc without pulmonary complications. Controls: n = 11; PAH: n = 14; ILD: n = 17; SSc no PAH no ILD: n = 27.

Initially, we investigated the differences in the expression levels of 37 EVs’ surface epitopes between SSc patients and the controls. The results are presented in the heat map in Figure 1.

**Figure 2:**
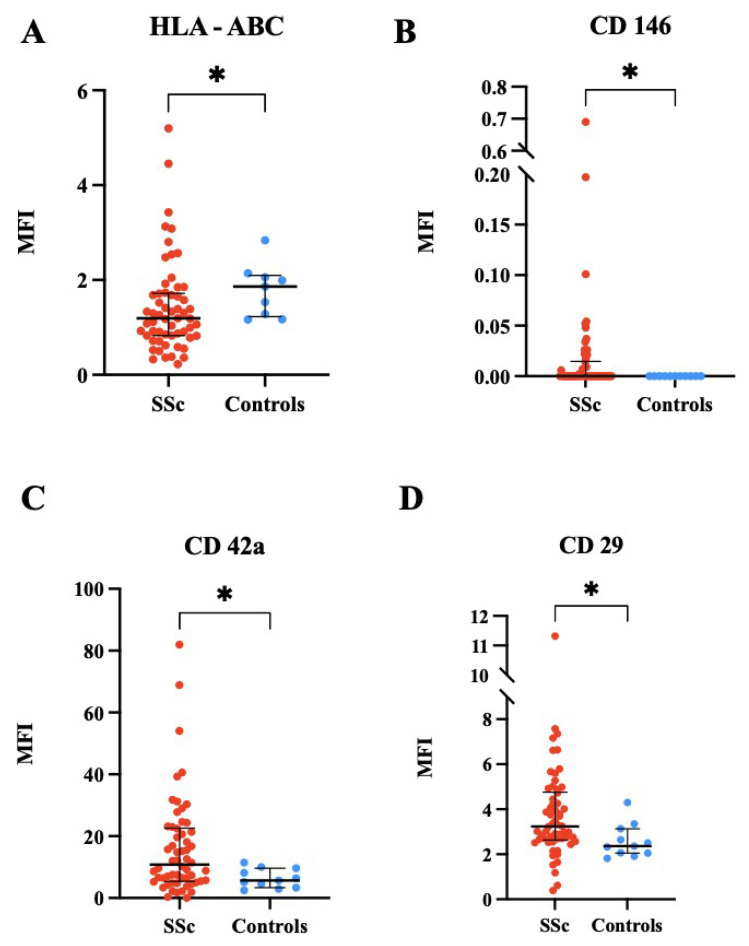
(**A**) HLA-ABC (human leukocytes antigen class 1 HLA-A, HLA-B, HLA-C) expression comparison between SSc patients and controls (* *p* = 0.02). (**B**) CD146 expression comparison between SSc patients and controls (* *p* = 0.03). (**C**) CD42a expression comparison between SSc patients and controls (* *p* = 0.02). (**D**) CD29 expression comparison between SSc patients and controls. (* *p* = 0.01). Values are expressed as median and [IQR]. * = *p* ≤ 0.05. MFI = mean fluorescence intensity. SSc: n = 58; controls: n = 11.

This first analysis indicated that CD146, CD42a, and CD29 had higher expression levels in patients with respect to the healthy controls (*p* = 0.03, 0.025, and 0.013, respectively). Conversely, HLA-ABC expression was higher in healthy subjects compared to patients, with a *p*-value of 0.0232. The results which reached statistical significance are shown in Figure 2. All markers analyzed are shown in Supplementary Table S2.

Furthermore, we established a multivariate model to evaluate if statistically significant markers in the univariate analysis would maintain a correlation with SSc in the multivariate analysis adjusted for demographic variable (age). After the multivariate analysis, only CD42a maintained its predictive role (*p* = 0.0478), as shown in Table 3.

### 2.2. Extracellular Vesicle Surface Epitopes Associated with SSc-PAH, SSc-ILD, and SSc No PAH No ILD

We then evaluated the differential expression of EV surface epitopes between SSc subgroups of the disease, aiming to find differences among PAH and ILD patients and those without pulmonary complications (SSc no PAH no ILD).

**Figure 3:**
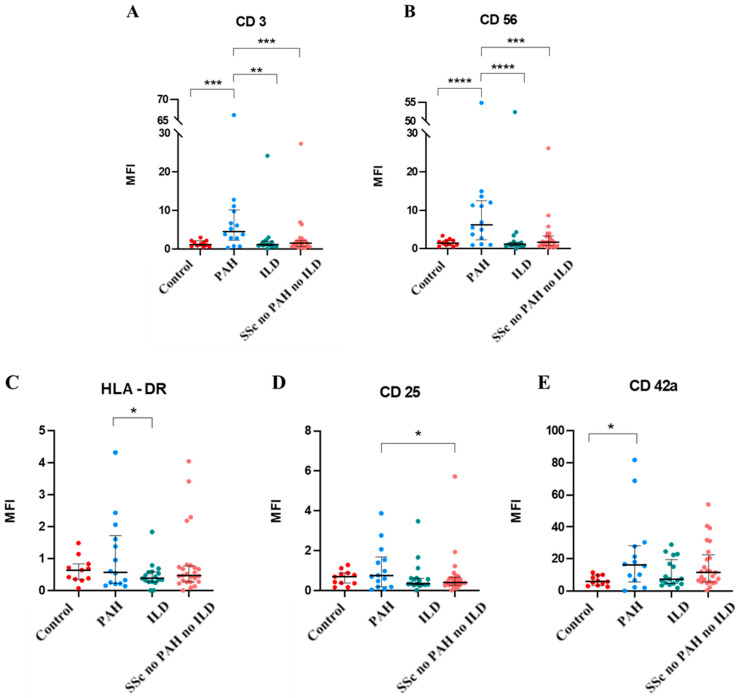
Association between CD3 expressions between disease subgroups and controls, ** *p* = 0.0133, *** *p* = 0.0033 (**A**). Association between CD56 expressions between disease subgroups and controls, *** *p* = 0.0016, **** *p* < 0.0001 (**B**). Association between HLA-DR expressions between disease subgroups and controls, * *p* < 0.05 (**C**). Association between CD25 expressions between disease subgroups and controls, * *p* < 0.05 (**D**). Association between CD42a expression between disease subgroups and controls. * *p* = 0.03 (**E**). Values are expressed as median and [IQR]. MFI = mean fluorescence intensity. Control: n = 11; PAH: n = 14; ILD: n = 17; SSc no PAH no ILD: n = 27.

The results of EV epitopes expression in the disease subgroups are reported in Supplementary Table S3, while those with significant differences (CD3, CD56, HLA-DR, CD25, and CD42a) are as shown in Figure 3. CD3 and CD56 were highly expressed in PAH EVs with respect to all other groups.

After the univariate analysis, we built a multivariate model to evaluate if these differential expressions were retained after correction for demographic (age) and disease-related (glomerular filtration rate, and steroid therapy) variables that were not homogeneous among groups. As shown in Table 4, only CD3 proved to be highly expressed after the multivariate analysis.

## 3. Discussion

Systemic sclerosis (SSc) represents a complex autoimmune disorder characterized by dysregulated immune responses, microvascular damage, and excessive tissue fibrosis affecting multiple organs, notably the skin and lungs [13,14]. The disease manifests with a spectrum of symptoms, ranging from skin thickening to severe complications such as interstitial lung disease (ILD) and pulmonary arterial hypertension (PAH), which significantly impact patient morbidity and mortality [15]. Despite advancements in understanding SSc pathogenesis, identifying reliable biomarkers for the early detection and prognosis of pulmonary complications remains a critical clinical challenge [16].

This study aimed to explore extracellular vesicle (EV) markers as potential non-invasive biomarkers for pulmonary complications in SSc patients. EVs, including exosomes and microvescicles, are recognized as essential mediators of intercellular communication, transporting bioactive molecules, such as proteins and nucleic acids, in autoimmune diseases like SSc [18]. The utility of EVs as disease biomarkers reflecting cellular processes relevant to disease pathogenesis still needs to be elucidated [17]. Meanwhile, the precise role of EVs is increasingly recognized.

In our cohort, we enrolled 58 patients diagnosed with SSc, categorized into subgroups, including 14 with pulmonary arterial hypertension (PAH), 17 with interstitial lung disease (ILD), and 27 with SSc without pulmonary complications (SSc no PAH no ILD). Additionally, 11 healthy subjects (controls) matched for sex and age were included for comparative analysis.

The demographic and clinical profiles of our SSc cohort generally aligned with the existing literature, with some notable differences observed. Specifically, the prevalence of PAH in our cohort (24% of SSc patients) exceeded epidemiological estimates, suggesting a potentially more severe disease phenotype in our study population [13,14]. Raynaud’s phenomenon was universally present. It is known that Raynaud’s syndrome causes spasms in small blood vessels in your fingers and toes. This limits blood flow and leads to symptoms like skin color changes, cold skin, and a pins-and-needles sensation. Common triggers of Raynaud’s attacks include cold weather and stress (100%), while interstitial pulmonary fibrosis (29%) was the predominant pulmonary manifestation, consistent with reported ranges [6,7,15]. These findings underscore the diverse clinical spectrum and organ involvement in SSc, necessitating tailored diagnostic and therapeutic approaches.

The comparative analysis among subgroups (PAH, ILD, and SSc no PAH no ILD) highlighted distinct clinical and hematological characteristics. Consistent with previous studies, our findings confirmed associations between limited cutaneous SSc (lcSSc), CENP antibodies, and the presence of pulmonary arterial hypertension [18]. Furthermore, PAH patients exhibited a significant reduction in glomerular filtration rate, potentially influenced by disease duration and advanced age [17].

Our investigative panel was focused on markers and clusters of differentiation (CD) pivotal in immune responses, including lymphocyte markers (CD3, CD56), neo-angiogenic markers (CD105), antigen presentation markers (HLA-ABC, HLA-DR), platelet activation markers (CD42a), and markers associated with neuronal processes [22,23].

The initial analysis aimed to identify differences in EVs marker expression between SSc patients and healthy controls. Our findings indicated elevated levels of specific EV markers such as CD146, CD42a, and CD29 in SSc patients compared to the controls [19,23,24]. HLA-ABC molecules, integral to major histocompatibility complex class I, are implicated as genetic susceptibility factors in autoimmune diseases, although their specific role in SSc remains under investigation [5]. CD146, recognized for its involvement in inflammatory processes and endothelial dysfunction, was significantly upregulated, consistent with its role in SSc-related vascular pathology [6]. Similarly, CD42a, predominantly expressed on platelets, and CD29, involved in tissue repair and fibrosis, underscored dysregulated processes contributing to tissue damage in SSc [2,3].

The multivariate analysis identified CD42a as being significantly correlated with PAH in SSc patients, suggesting its potential as a diagnostic biomarker for this complication. This finding aligns with studies demonstrating elevated levels of platelet-derived microparticles, characterized by CD42a expression, in PAH pathogenesis [25]. However, further research is needed to validate CD42a as a specific biomarker for PAH in SSc and elucidate underlying pathogenic mechanisms.

Following this initial analysis focusing on differences in EV surface epitope expression between SSc patients and healthy controls, we explored if there were statistically significant associations between epitope expression and specific disease subgroups. In the univariate analysis, CD3, HLA-DR, CD25, and CD42a exhibited significantly higher levels in PAH patients compared to the other groups.

Notably, CD3, a marker of T lymphocytes, demonstrated significant overexpression in PAH patients compared to the other subgroups and controls together with CD56. This finding suggests a potential role for specific T-cell subsets, including CD3^+^CD56^+^ natural killer T (NKT) cells, in the pathogenesis of PAH within the context of SSc [1,4]. Previous research has implicated NKT cells in vascular remodeling and inflammation, mechanisms central to PAH progression [9,10]. The elevated expression levels of CD3^+^ and CD56 in pulmonary arterial hypertension associated with systemic sclerosis (SSc-PAH) endothelial vessels suggest potential co-expression of these markers, indicative of a distinct subset of infiltrating NK lymphocytes within the vascular milieu. This observation prompts further investigation to elucidate the functional implications of this co- expression in the pathogenesis of SSc-PAH. NK lymphocytes, characterized by the concurrent expression of CD3 and CD56 markers, are known for their potent cytotoxic and regulatory roles in immune responses. In the context of SSc-PAH, their presence within endothelial vessel walls underscores a probable involvement in vascular remodeling and inflammatory processes central to disease progression. Understanding the specific functions and interactions of these CD3^+^/CD56^+^ NK lymphocytes in SSc-PAH pathophysiology is crucial for delineating their contributions to endothelial dysfunction and vascular pathology [22,26,27]. Our data, taken as a whole, might suggest a double role for EVs. First, early detection of PAH via the CD pattern of EVs might help in early precise pharmacological choice; second, pharmacological manipulation of EV release or CD expression might have a therapeutic role in the management of this complex pathology.

Despite its promising findings, our study possesses limitations such as a small sample size and a single-center design, which may affect the generalizability of the results. Additionally, the heterogeneity of SSc and its pulmonary complications underscores the need for larger, multicenter studies to validate these findings across diverse patient populations [28,29]. SSc autoimmune disease has an adult female predominance. The overall female-to-male ratio is usually 3:1 or greater. Furthermore, female patients most frequently develop serious complications. In our clinical population, male patients were not present; thus, it was not possible to include them [30]. A possible bias of our study is represented by the average age of the patients. In fact, all patients included in the study were over the age of 59. SSc is a pathology that affects elderly individuals, and these patients represent the typical patient in our area. Future research should focus on elucidating the specific mechanisms by which EV markers, such as CD3 and CD56, contribute to PAH complications in SSc, and as therapeutic targets aimed at modulating NK cell-mediated immune responses within affected endothelial microenvironments [27]. Our results indicate that EV epitopes could be used as integrative biomarkers to evaluate disease progression from SSc to SSc PAH. Unfortunately, our population number is limited, since the pathology is fortunately quite rare. Furthermore, the data presented are intended to be integrative to those obtained by routine tests performed in clinical practice for the evaluation of disease progression. The advantage of our experimental approach is that no extraction of EVs from a liquid biopsy is needed. EV epitopes are analyzed directly from serum, thus avoiding extraction bias. Future aims of these kinds of experimental approaches could be the evaluation of the possible progression of the diseases by analyzing the variation dynamics of the epitopes in larger cohort of patients. Our work, even though it could appear as a description of epitopes expression on the EVs of patients, taken as a whole, could contribute to suggesting new approaches for targeted therapies for these serious pathologies.

## 4. Material and Methods

### 4.1. Patients

In this prospective cohort study, we enrolled 58 patients from October 2016 to December 2019 with a diagnosis of SSc who were periodically referred to the tertiary level outpatient clinic of the Rheumatology department of “Maggiore della Carità” University Hospital (Novara, Italy) and to the Pulmonary Hypertension Outpatient Clinic of the Cardiological Department of “Maggiore della Carità” University Hospital (Novara, Italy). The patient cohort was sourced from the “Novara Bio-bank” registry, approved by the Ethics Committee of the AOU Maggiore della Carità, Novara, on 9 September 2016 (CE:108/16).

The study population was divided into three groups based on the manifestation of disease complications: (1) the pulmonary arterial hypertension (PAH) group; (2) the interstitial lung disease (ILD) group; and (3) the group without PAH and ILD (SSc no PAH no ILD). The diagnosis of PAH was determined based on mean pulmonary artery pressure values ≥ 25 mmHg, with capillary wedge pressure (WEDGE) < 15 mmHg and pulmonary vascular resistance >3 Wood units. Interstitial lung disease diagnosis relied on clinical assessment, HRCT, and pulmonary function tests, specifically DLCO corrected for Hb < 80%. Patients in the third group did not meet the diagnostic criteria of the first two categories. Out of the 58 patients in the study, 54 were diagnosed with systemic sclerosis (SSc), and only 4 were classified under scleroderma overlap syndrome. However, clinically, these patients were included in the group named “SSc no PAH no ILD”. Additionally, 11 healthy controls, matched for sex and age, were included.

The study’s inclusion criteria comprised individuals aged 18 years or older, with a confirmed diagnosis of systemic sclerosis (SSc) adhering to the ACR/EULAR 2013 diagnostic criteria, encompassing both scleroderma syndrome and scleroderma overlap syndrome. The exclusion criteria were refusal to provide informed consent, an inability to undergo the checks required by the protocol, an age under 18 years, and pregnancy.

Patients were treated following the international guidelines to ensure the best therapeutic options. The drugs used were Phosphodiesterase type-5 (PDE-5) inhibitors, glucocorticoids, methotrexate (MTX), hydroxychloroquine (HCQ), intravenous prostanoid, calcium antagonists, and endothelin receptor antagonists (ERAs). The therapies are detailed in Table 2.

#### 4.1.1. Ethical Committee

The study protocol was approved by the local ethical committee (Comitato Etico Interaziendale di Novara, Protocol n° CE 108/16) and conducted in strict accordance with the principles of the Declaration of Helsinki. Informed consent was obtained from all participants in the study.

#### 4.1.2. Clinical Evaluation

The clinical evaluation involved a review of patients’ medical history and a physical examination. This included a 12-lead ECG recording limb and precordial leads at a standard paper speed of 25 mm per second; chest X-rays in anteroposterior and side views; and lung function tests (LFTs) performed using a spirometer (COSMED, Rome, Italy) with standardized equipment and methods. The spirometer was connected to a computer system running “Medisoft Expair 1.28.20” software. The evaluated parameters included total lung capacity (TLC), forced expiratory volume in 1 s (FEV1), and the FEV1/TLC ratio (also known as the Tiffeneau–Pinelli index). Additionally, lung diffusion capacity for carbon monoxide (DLCO) was measured using the single-breath technique according to Jones–Meade. Transthoracic echocardiograms (TTE) utilized GE Medical Systems’ Vivid 7 or E9 ultrasound systems (Horten, Norway) with a 1.7/3.4 MHz harmonic imaging probe. The procedures adhered to standardized positions outlined in the American Society of Echocardiography guidelines and were performed by a TTE specialist with expertise in pulmonary hypertension. The assessed parameters included peak systolic pressure in the pulmonary artery (sPAP), the size of the right atrium (RAA), the dimension of the right ventricle (RVD), and the ejection fraction (EF) of the heart. The systolic performance of the right ventricle was assessed by measuring the tricuspid annular plane systolic excursion (TAPSE). Patients suspected of PAH underwent right heart catheterization (RHC) within one month of the TTE, following international protocols. PAH was diagnosed with a mean pulmonary artery pressure (mPAP) ≥ 25 mmHg, pulmonary capillary wedge pressure ≤ 15 mmHg, and pulmonary vascular resistance > 3 Wood units. In cases where RHC was contraindicated, pulmonary hypertension was diagnosed based on an estimated sPAP ≥ 35 mmHg from echocardiography and other criteria indicating a high likelihood, consistent with the 2015 ESC/ESR guidelines.

### 4.2. Laboratory Analyses

Blood samples were collected in EDTA tubes from each subject, centrifuged at room temperature for 10 min at 3000 rpm within one hour of collection, and subsequently stored at −80 °C until the analyses. The laboratory evaluation included the following: blood count, creatininemia, urine test, uricemia, liver function indices, erythrocyte sedimentation rate (ESR), C-reactive protein (CRP), complement C3 and C4, brain natriuretic peptide (BNP), and thyroid-stimulating hormone (TSH). In addition, immunity typing was performed by detecting antibodies to the Antinuclear Antibody (ANA), anti-Ro autoantibodies directed against a ribonucleoprotein consisting of an RNA molecule and two proteins of 60 and 52 KD (anti-Ro-60 and anti-Ro-52), anti-La (autoantibodies against nuclear 47 kD phosphoprotein), anti-Scl70 (antibody against anti-DNA Topoisomerase 1), anti-centromere antibodies (CENP), RF (rheumatoid factor) AMA (anti-mitochondrion), anti-RNP antibodies, brain natriuretic peptide (BNP), and antiphospholipid antibodies (LAC, anticardiolipin, anti-β2-glycoprotein).

The results of LAC functional tests, like silica clotting time (SCT) and dilute Russel’s viper venom test (DRVVT), as well as tests for anti-cardiolipin and anti-β2-glycoprotein antibodies (IgG and IgM), are not included in Table 2, as they were negative.

Procedures for the clinical, laboratory, and instrumental evaluation of patients with SSc were already described in previous papers [31,32,33].

#### Extracellular Vesicle Characterization

The characterization of serum EVs was performed using a MACSplex kit (MACSplex exosome kit, Human, Miltenyi Biotec, Auburn, CA, USA) following the manufacturer’s instructions. A MACSPlex kit comprises a cocktail of various fluorescently labeled bead populations, each coated with a specific antibody binding the respective surface epitopes. The 39 bead populations can be distinguished by different fluorescence intensities via flow cytometry. Briefly, 1 × 10^9^ EVs were determined by nanoparticle tracking analysis (NTA), and later, were diluted in MACSPlex buffer (MPB) to obtain a final volume of 120 µL. This mixture was then combined with 15 µL of MACSPlex Exosome Capture Beads and incubated overnight at 4 °C with gentle shaking, protected from exposure to light. Following incubation, the EVs bead complexes were washed by adding 500 µL of MACSplex buffer, followed by centrifugation at 3000× *g* for 5 min at room temperature. The supernatant (for a total of 500 µL) was carefully removed, and 5 µL each of anti-CD9, anti-CD63, and anti-CD81 antibodies conjugated with APC were added to each sample. After an hour of incubation with gentle agitation at room temperature, 500 µL of MACSplex buffer was added, and the solution was subsequently centrifuged at 3000× *g* for 5 min. After final centrifugation, 350 µL of the supernatant was aspirated, leaving the residual volume to resuspend the pellet. Sample analysis was conducted via flow cytometry using the Celesta FACS system (BD Biosciences; Franklin Lakes, NJ, USA). The background signals derived from the MACSplex buffer and isotype controls (recombinant engineered antibody, REA, or mouse IgG) were subtracted from the sample data. The resulting values were normalized to the median fluorescence intensity (MFI) of the tetraspanins (CD9, CD63, CD81) and presented as percentage values.

### 4.3. Statistical Analysis

For continuous variables, the measures of centrality and dispersion were medians and interquartile ranges [IQR], and comparisons between groups regarding these variables were performed using the Mann–Whitney U test and the Kruskal–Wallis test. The Pearson χ^2^ test was used to analyze the association between categorical variables, and the results are shown as frequencies (%). Correlations were performed with the Spearman’s rank correlation coefficient and linear regression for significant predictors in the univariate model. Multivariate regression models were built to identify the variables independently associated with the severity score. The threshold for statistical significance was 0.05 (two-tailed). Statistical analyses were performed with Stata statistical software version 17.0 (Stata Corp, 4905 Lakeway Drive College Station, TX, USA), while graphs were created using GraphPad Prism version 9.4.0 (GraphPad Software, La Jolla, CA, USA).

## 5. Conclusions

In conclusion, our study contributes novel insights regarding the potential of EV markers in the early detection of PAH-complicated SSc. EV markers can contribute to more personalized medicine, especially for more personalized drug therapy. Our data and technical approach to patients’ samples have the advantage of low bias due to the manipulation of liquid biopsy. The direct characterization of EV epitopes, combined with other commonly recognized routine tests, might represent a new tool for the early diagnosis of PAH-related complications and possibly provide a more personalized pharmacological approach.

## Figures and Tables

**Table 1:** Demographic, clinical, and laboratory features of the study SSc population (continuous variables). Median data are reported with interquartile ranges [IQR]. WBCs = white blood cells; Hb = hemoglobin; GFR = glomerular filtration rate; RDW = red blood cell distribution width; PLTS = platelets; PAH = pulmonary arterial hypertension; ILD = interstitial lung disease; SSc no PAH no ILD = scleroderma without pulmonary complications; KW = Kruskal–Wallis test. *p* = *p*-value. In Post Hoc test: 1 = PAH; 2 = ILD; 3 = SSc without pulmonary complications (SSc no PAH no ILD).

Variable	PAH	ILD	SSc No PAH No ILD	Anova KW	*p*	Post Hoc
Age of enrolment (years)	77.5 [71.0–82.0]	66.0 [62.0–71.0]	67.0 [59.0–78.0]	76.075	0.0223	1 vs. 2, *p* = 0.0304; 1 vs. 3, *p* = 0.0584
Disease duration (years)	12.0 [7.0–14.0]	12.0 [6.0–18.5]	10.0 [4.0–14.0]	11.540	0.5616	
Hb (g/dL)	12.0 [11.10–3.30]	11.3 [10.30–12.25]	13.0 [12.20–3.90]	109.164	0.0043	2 vs. 3, *p* = 0.0035
GFR (mL/min)	58 [45.00–64.00]	91 [54.00–101.00]	89 [57.00–99.00]	102.998	0.0058	1 vs. 2, *p*= 0.0231; 1 vs. 3, *p*= 0.0085
RDW (%)	15.3 [13.90–16.60]	14.4 [13.30–15.40]	13.6 [12.35–14.15]	8.4534 *p* = 0.0146		1 vs. 3, *p* = 0.0112
WBCs (×10^3^/μL)	6.67 [5.77–7.61]	6.40 [5.34–7.64]	5.90 [4.85–6.67]	3.0701 *p* = 0.2155		
PLTS	209 [164.00–292.00]	269 [217.50–302.00]	254 [224.00–300.00]	3.2894 *p* = 0.1931		

**Table 2:** Demographic, clinical, and laboratory features of the study SSc population (categorical variables). Bold values are statistically significant. PAH = pulmonary arterial hypertension; SSc no PAH no ILD = scleroderma without pulmonary complications; HCQ = hydroxychloroquine; MTX = methotrexate; Ab anti-ANA+ = Antinuclear Anti-body; Ab anti-Ro60+ = autoantibodies directed against a ribonucleoprotein; Ab anti-Ro52+ = autoantibodies directed against a ribonucleoprotein; Ab anti-Scl70+ = Topoisomerase 1; Ab anti-CENP+ = anti-centromere antibodies; Ab anti-RNP+ = anti-ribonucleoprotein antibodies; Ab anti-AMA+ = anti-mitochondrion antibodies; ERA = endothelin receptor antagonists; PDE5-I = phosphodiesterase 5 inhibitors. *p* = *p*-value; χ^2^ = chi-square.

Variables	PAH (n = 14)	ILD (n = 17)	SSc No PAH No ILD (n = 27)	*p*	χ^2^
**Clinical features**					
Diffuse sclerosis	1	6	5	0.1457	3.853, df = 2
Limited sclerosis	12	8	22	**0.02**	7.822, df = 2
Digital ulcers	5	10	8	0.147	3.835, df = 2
Digestive tube involvement	3	10	13	0.1923	3.298, df = 2
Kidney involvement	0	1	0	0.2932	2.454, df = 2
Arterial hypertension	8	9	13	0.8551	0.313, df = 2
Cardiopathy	5	2	4	0.1798	3.432, df = 2
Liver disease	4	4	3	0.3405	2.155, df = 2
**Laboratory features**					
Ab anti-ANA+	13	16	23	0.9703	0.060, df = 2
Ab anti-Ro60+	1	2	2	0.8596	0.303, df = 2
Ab anti-Ro52+	3	5	1	0.0564	5.751, df = 2
Ab anti-La+	1	1	0	0.3984	1.841, df = 2
Ab anti-Scl70+	0	6	2	**0.0075**	9.775, df = 2
Ab anti-CENP+	12	5	11	**0.0043**	10.895, df = 2
Ab anti-RNP+	0	1	0	0.2932	2.454, df = 2
Ab anti-AMA+	1	3	0	0.0796	5.061, df = 2
**Drug Treatments**					
HCQ	6	9	12	0.8171	0.404, df = 2
Glucocorticoids	0	7	6	**0.0237**	7.488, df = 2
MTX	0	5	7	0.0867	4.891, df = 2
Calcium antagonists	4	8	13	0.4506	1.594, df = 2
Intravenous prostanoid	5	13	16	0.0719	5.266, df = 2
Endothelin receptor antagonist (ERA)	7	1	1	**0.0002**	16.777, df = 2
PDE5-I Phosphodiesterase type 5 (PDE-5) inhibitor	7	0	0	**0.0001**	25.020, df = 2

**Table 3:** Multivariate analysis corrected by age.

Predictor	Odds Ratio	Standard Error	χ^2^	*p*	95% Confidence Interval
CD42a	1.1396	0.066035	8.4038	0.0478	1.0012–1.2971

**Table 4:** Multivariate analysis corrected by demographic and disease-related variables.

Predictor	Odds Ratio	Standard Error	χ^2^	*p*	95% Confidence Interval
CD3	1.8278	0.18968	7.3946	0.0015	1.0012–1.2971

## Data Availability

The original contributions presented in this study are included in the article/Supplementary Material. Further inquiries can be directed to the corresponding author.

## Supplementary Materials

The following supporting information can be downloaded at: https://www.mdpi.com/article/10.3390/ph18020259/s1, Table S1. Data are reported with medians and interquartile ranges [IQRs]; Table S2. Comparison of the 37 epitopes expressed on EV surface between SSc patients and controls. Data are reported with medians and interquartile ranges [IQRs]; Table S3. This Table describes the analyses of variance of EVs membrane epitopes among the three subgroups of disease and healthy subjects.

## Abbreviations

EVs	Extracellular vesicles
ILD	Interstitial lung disease
PAH	Pulmonary arteriolar hypertension
SSc	Systemic sclerosis
