# Supplementary material for: Extracellular Vesicles as a Potential Biomarker of Pulmonary Arterial Hypertension in Systemic Sclerosis
Source: Pharmaceuticals (Basel). 2025 Feb 14;18(2):259. doi: 10.3390/ph18020259 (PMC11859480; doi:10.3390/ph18020259)
Supplement: Supplementary file 1 [file pharmaceuticals-18-00259-s001.zip › pharmaceuticals-3445273-supplementary.pdf]

Supplementary Materials

Supplementary Table S1. Data are reported with medians and interquartile ranges [IQR]. WBC = white blood cells; Hb = hemoglobin; RDW = red blood cell distribution width; PLTS = platelets; PAH = pulmonary arterial hypertension, SSc = scleroderma without pulmonary complications; KW = Kruskal Wallis test. In Post Hoc: 1 = PAH; 2 = interstitial lung disease (ILD); 3 = SSc without pulmonary complications (SSC no PAH no ILD). Statistically significant values are **evidenced** in bold.

**Variables**	**PAH**	**ILD**	**SSc**	**Anova KW**	***p* value**	**Post Hoc**
Age at withdrawal (years)	77.5 [71.0-82.0]	66.0 [62.0-71.0]	67.0 [59.0-78.0]	7.6075	**0.0223**	1 *vs.* 2 **0.0304** 1*vs* 3 **0.0584**
Length of illness (years)	12.0 [7.0-14.0]	12.0 [6.0-18.5]	10.0 [4.0-14.0]	1.1540	0.5616	
WBC ($\times 10^3/\mu L$)	6.67 [5.77-7.61]	6.40 [5.34-7.64]	5.90 [4.85-6.67]	3.0701	0.2155	
Hb (g/dL)	12.0 [11.10-13.30]	11.3 [10.30-12.25]	13.0 [12.20-13.90]	10.9164	**0.0043**	2 *vs.* 3 0.0035
RDW **(%)**	15.3 [13.90-16-60]	14.4 [13.30-15.40]	13.6 [12.35-14.15]	8.4534	**0.0146**	1 *vs.* 3 **0.0112**
PLTS	209 [164.00-292.00]	269 [217.50-302.00]	254 [224.00-300.00]	3.2894	0.1931	
Filtrate (mL/min)	58 [45.00-64.00]	91 [54.00-101.00]	89 [57.00-99.00]	10.2998	**0.0058**	1 *vs.* 2 **0.0231** 1 *vs.* 3 **0.0085**

Supplementary Table S2. Comparison of the 37 epitopes expressed on E*Vs* surface between SSc patients and controls. Data are reported with medians and interquartile ranges [IQR]. Statistically significant results are shown in bold. The values are expressed in median fluoresce intensity (MFI) and adjusted by the tetraspanins (CD9, CD63 and CD81).

**Epitopes**	**SSc**	**Controls**	**Z**	***p***
CD3	1.63 [0.86-3.05]	1.22 [0.80-2.20]	0.79501	0.4266
CD4	0.00 [0.00-0.02]	0.00 [0.00-0.00]	1.11362	0.2654
CD19	0.00 [0.00-0.03]	0.00 [0.00-0.01]	1.41242	0.1578
CD8	0.06 [0.00-0.19]	0.06 [0.02-0.08]	0.44445	0.6567
HLA-DR	0.47 [0.27-0.73]	0.64 [0.35-0.83]	-0.58194	0.5606
CD56	1.74 [0.94-4.14]	1.47 [0.92-2.13]	1.00811	0.3134
CD105	0.27 [0.15-0.46]	0.27 [0.18-0.39]	-0.04918	0.9607
CD2	0.05 [0.02-0.16]	0.10 [0.04-0.19]	-1.15952	0.2462
CD1c	0.00 [0.00-0.04]	0.00 [0.00-0.05]	0.48862	0.6251
CD1c	0.48 [0.27-0.75]	0.68 [0.36-0.87]	-0.61470	0.5387
CD49e	3.50 [2.33-6.13]	2.46 [2.09-3.41]	1.45069	0.1469
ROR1	0.04 [0.01-0.24]	0.02 [0.00-0.05]	1.68888	0.0912
CD209	0.02 [0.00-0.10]	0.01 [0.00-0.01]	1.20934	0.2265
CD9	1.89 [1.15-2.09]	1.75 [1.66-1.82]	0.23768	0.8121
SSEA-4	0.05 [0.03-0.08]	0.08 [0.05-0.15]	-1.36334	0.1728
HLA-ABC	1.19 [0.83-1.71]	1.86 [1.28-2.06]	-2.27029	**0.0232**
CD63	0.68 [0.51-0.90]	0.81 [0.79-0.96]	-0.93435	0.3501
CD40	2.93 [1.97-4.63]	2.60 [2.30-3.24]	0.36882	0.7123
CD62P	7.52 [3.55-16.72]	4.07 [2.68-7.68]	1.54904	0.1214

**Epitopes**	**SSc**	**Controls**	**Z**	***p***
CD11c	0.02 [0.00-0.06]	0.03 [0.01-0.05]	-0.94282	0.3458
CD81	0.41 [0.24-0.76]	0.40 [0.38-0.50]	0.31966	0.7492
MCSP	0.00 [0.00-0.02]	0.00 [0.00-0.00]	1.83584	0.0664
CD146	0.00 [0.00-0.01]	0.00 [0.00-0.00]	2.16581	**0.0303**
CD41b	2.35 [1.06-2.82]	2.50 [2.12-3.03]	-0.84419	0.3986
CD42a	10.79 [5.47-22.47]	5.70 [3.35-9.68]	2.23750	**0.0253**
CD24	0.04 [0.01-0.11]	0.03 [0.02-0.05]	0.40193	0.6877
CD86	0.00 [0.00-0.02]	0.00 [0.00-0.02]	1.05024	0.2936
CD44	0.08 [0.03-0.19]	0.11 [0.06-0.14]	-0.54999	0.5823
CD326	0.01 [0.00-0.10]	0.01 [0.00-0.02]	1.54523	0.1223
CD133-1	0.01 [0.00-0.07]	0.00 [0.00-0.01]	1.66955	0.0950
CD29	3.23 [2.66-4.73]	2.36 [2.06-3.13]	2.48338	**0.0130**
CD69	0.46 [0.24-0.72]	0.67 [0.52-0.78]	-1.30321	0.1925
CD142	0.07 [0.04-0.19]	0.06 [0.04-0.25]	0.29506	0.7679
CD45	0.52 [0.29-1.03]	0.75 [0.37-1.13]	-0.71305	0.4758
CD31	2.79 [1.23-4.21]	2.26 [1.84-2.43]	0.92616	0.3544
CD20	0.00 [0.00-0.03]	0.00 [0.00-0.00]	1.43087	0.1525
CD14	0.01 [0.00-0.04]	0.01 [0.00-0.04]	-0.19261	0.8473

Supplementary Table S3. This Table describes the analyses of variance of E*Vs* membrane epitopes among the three subgroups of disease and healthy subjects. For Post Hoc analysis 0 = healthy subjects, 1 = PAH, 2 = ILD, 3 = SSc no PAH no ILD. Statistically significant values are evidenced in bold.

**Variable**	**PAH (14)**	**ILD (17)**	**SSc no PAH no ILD (27)**	**Controls (11)**	**KW *p* value**	**Post Hoc**
CD3	6.31 [3.81-9.77]	1.18 [0.93-2.14]	1.11 [0.71-2.08]	1.22 [0.80-2.20]	18.0017 *p* =**0.0004**	0*vs.*1 0.0133<br>1*vs.*2 0.0033<br>1*vs.*3 0.0005
CD4	0.00 [0.00-0.01]	0.00 [0.00-0.00]	0.01 [0.00-0.04]	0.00 [0.00-0.00]	3.3027 *p* =0.3473	
CD19	0.00 [0.00-0.03]	0.00 [0.00-0.05]	0.00 [0.00-0.02]	0.00 [0.00-0.08]	2.0462 *p* =0.5629	
CD8	0.08 [0.02-0.21]	0.04 [0.00-0.08]	0.06 [0.01-0.24]	0.06 [0.02-0.08]	2.2912 *p* =0.5142	
HLA-DR	1.28 [0.50-2.19]	0.34 [0.25-0.53]	0.45 [0.27-0.69]	0.64 [0.35-0.83]	9.6182 *p* =**0.0221**	1*vs.*2 0.0162
CD56	7.74 [4.95-3.58]	1.14 [0.81-1.85]	1.65 [0.90-2.71]	1.47 [0.92-2.13]	25.5031 *p* =**0.002**	0*vs.*1 0.0016<br>1*vs.*2 0.0000<br>1*vs.*3 0.0002
CD105	0.26 [0.12-0.37]	0.29 [0.21-0.39]	0.27 [0.12-0.60]	0.27 [0.18-0.39]	0.3778 *p* =0.9448	
CD2	0.22 [0.00-0.31]	0.05 [0.01-0.09]	0.05 [0.03-0.12]	0.10 [0.04-0.19]	3.4749 *p* =0.3240	
CD1c	0.00 [0.00-0.03]	0.00 [0.00-0.05]	0.01 [0.00-0.03]	0.00 [0.00-0.05]	0.4046 *p* =0.9393	
CD25	1.31 [0.46-2.06]	0.42 [0.30-0.59]	0.37 [0.24-0.61]	0.68 [0.36-0.87]	9.0804 *p* =0.0282	1*vs.*3 0.0253
CD49e	6.39 [2.32-11.53]	2.75 [2.33-4.07]	3.45 [2.38-5.78]	2.46 [2.09-3.41]	6.9899 *p* =0.0722	
ROR1	0.09 [0.01-0.65]	0.04 [0.01-0.38]	0.03 [0.00-0.09]	0.02 [0.00-0.05]	6.5695 *p* =0.0870	
CD209	0.00 [0.00-0.10]	0.03 [0.00-0.01]	0.02 [0.00-0.11]	0.01 [0.00-0.01]	3.1929 *p* =0.3628	
CD9	1.06 [0.64-1.93]	1.91 [1.70-2.07]	1.94 [1.58-2.19]	1.75 [1.66-1.82]	5.5401 *p* =0.1363	
SSEA-4	0.09 [0.04-0.26]	0.05 [0.01-0.08]	0.04 [0.01-0.08]	0.08 [0.05-0.15]	8.3342 *p* =.0396	
HLS-ABC	1.17 [0.78-3.08]	1.18 [0.83-1.39]	1.19 [0.83-1.68]	1.86 [1.28-2.06]	6.4491 *p* =0.0917	
CD63	0.83 [0.37-1.46]	0.74 [0.63-1.00]	0.61 [0.48-0.88]	0.81 [0.79-0.96]	4.2836 *p* =.2324	
CD40	4.90 [0.93-9.80]	2.97 [2.25-3.27]	2.30 [1.84-4.60]	2.61 [2.30-3.24]	2.2175 *p* =0.5285	
CD62*P*	16.36 [5.86-30.07]	5.66 [5.08-9.08]	6.65 [3.03-16.26]	4.07 [2.68-7.68]	6.1440 *p* =0.1048	
CD11c	0.01 [0.00-0.12]	0.02 [0.00-0.03]	0.01 [0.00-0.11]	0.03 [0.01-0.05]	0.9575 *p* =0.8115	
CD81	0.76 [0.57-1.04]	0.34 [0.24-0.49]	0.34 [0.21-0.69]	0.40 [0.38-0.50]	7.6171 *p* =0.0546	
MCS*P*	0.00 [0.00-0.01]	0.00 [0.00-0.05]	0.00 [0.00-0.017]	0.00 [0.00-0.00]	5.4437 *p* =0.1420	
CD146	0.00 [0.00-0.01]	0.00 [0.00-0.02]	0.00 [0.00-0.02]	0.00 [0.00-0.00]	5.0744 *p* =0.1664	

Variable	PAH (14)	ILD (17)	SSc no PAH no ILD (27)	Controls (11)	KW *p* value	Post Hoc
CD41b	1.48 [0.99-2.64]	2.33 [1.67-2.55]	2.61 [1.04-3.51]	2.50 [2.12-3.03]	2.6052 *p* =0.4566	
CD42a	16.21 [10.14-30.36]	6.54 [4.27-16.91]	9.59 [5.74-24.36]	5.70 [3.35-9.68]	9.7432 *p* =**0.0209**	0*vs.*1 0.0302
CD24	0.04 [0.01-0.16]	0.04 [0.01-0.06]	0.05 [0.01-0.11]	0.03 0.02-0.05]	0.4384 *p* =0.9322	
CD86	0.00 [0.00-0.01]	0.00 [0.00-0.03]	0.00 [0.00-0.01]	0.00 [0.00-0.02]	2.0256 *p* =0.5671	
CD44	0.19 [0.01-0.35]	0.04 [0.02-0.09]	0.08 [0.03-0.14]	0.11 [0.06-0.14]	4.2844 *p* =0.2323	
CD326	0.1 [0.00-0.42]	0.01 [0.00-0.10]	0.001 [0.00-0.06]	0.00 [0.00-0.02]	6.4325 *p* =0.0924	
CD133-1	0.03 [0.00-0.32]	0.01 [0.00-0.07]	0.01 [0.00-0.05]	0.00 [0.00-0.01]	3.7950 *p* =0.2845	
CD29	4.01 [2.16-4.99]	3.25 [2.69-3.75]	2.99 [2.55-4.87]	2.36 [2.06-3.13]	6.3648 *p* =0.0952	
CD69	0.35 [0.13-0.62]	0.57 [0.31-0.74]	0.48 [0.26-0.67]	0.67 [0.52-0.78]	3.1525 *p* =0.3687	
CD142	0.16 [0.03-0.37]	0.07 [0.06-0.20]	0.08 [0.03-0.12]	0.06 [0.04-0.22]	1.3616 *p* =0.7146	
CD45	1.54 [0.18-2.34]	0.40 [0.36-0.63]	0.52 [0.29-0.71]	0.75 [0.37-1.13]	3.6575 *p* =0.3009	
CD31	1.51 [0.37-5.54]	2.83 [1.81-4.21]	2.94 [1.31-4.15]	2.26 [1.84-2.43]	2.9448 *p* =0.4002	
CD20	0.00 [0.00-0.05]	0.01 [0.00-0.03]	0.00 [0.00-0.01]	0.00 [0.00-0.00]	4.4472 *p* =0.2170	
CD14	0.01 [0.00-0.04]	0.01 [0.00-0.04]	0.01 [0.00-0.04]	0.01 [0.00-0.04]	0.4245 *p* =0.9351	
